# Revisiting the associations between cooking oils and survival among older people in China: A nationwide, community-based, prospective cohort study

**DOI:** 10.1371/journal.pone.0344282

**Published:** 2026-03-05

**Authors:** Kexin Wang, Chao Ban, Liming Zhao, Haiyan Ruan, Ziqiong Wang, Yi Zheng, Sen He

**Affiliations:** 1 Department of Cardiology, West China Hospital, Sichuan University, Chengdu, China; 2 Department of Equipment, West China Hospital, Sichuan University, Chengdu, China; 3 Department of Cardiology, Hospital of Chengdu Office of People’s Government of Tibetan Autonomous Region, Chengdu, China; 4 Department of Cardiology, Hospital of Traditional Chinese Medicine, Shuangliu District, Chengdu, China; 5 Department of Cardiology, Karamay Hospital of Integrated Chinese and Western Medicine, Karamay, China; Health Sciences University Istanbul Training and Research Hospital, TÜRKIYE

## Abstract

**Background:**

The study aimed to investigate the associations between cooking oils and survival outcomes in a nationwide, community-based, prospective cohort study of older adults in China.

**Methods:**

A total of 5372 older participants (median age: 85.0, inter-quartile range [IQR] age: 77.0–93.0; male: 46.1%) from the 2014 wave of the Chinese Longitudinal Healthy Longevity Survey (CLHLS) in 2014 were included, with follow-up until 2018. The exposure was cooking oils, including vegetable oils and lard, and outcomes were overall survival (OS) and disease-specific survival (i.e., cardiovascular disease [CVD]-specific survival and non-CVD-specific survival). Accelerated failure time (AFT) models were used to analyze the associations between cooking oils and study outcomes.

**Results:**

During a median follow-up of 3.5 years (IQR: 2.4–4.2 years), 2064 (38.4%) deaths were recorded, including 433 CVD deaths, 1229 non-CVD deaths, and 402 deaths with unknown causes. Kaplan-Meier analysis revealed cooking with lard was associated a higher CVD-specific survival probability than vegetable oils (93.9% vs. 88.2%, log-rank p < 0.001); however, there were no significant differences in OS and non-CVD-specific survival between the two groups (log-rank p = 0.076 and 0.210, respectively). Furthermore, multivariate AFT models indicated cooking with lard was significantly associated with a longer CVD-specific survival compared to vegetable oils (time ratio [TR]=1.44, 95% confidence interval [CI]: 1.08–1.91), and was not associated with OS and non-CVD-specific survival, with adjusted TRs of 1.06 (95% CI: 0.95–1.18) for OS and 1.08 (95% CI: 0.93–1.26) for non-CVD-specific survival, respectively.

**Conclusions:**

Cooking with lard was associated with significantly longer CVD- specific survival compared to vegetable oils among older adults in China.

## 1. Background

Persistent health disparities continue to exist in the modern era [1,2], and factors associated with these disparities are complex and multifactorial, encompassing various elements such as lifestyles, comorbidities, and genetic predispositions [3–5]. Among these factors, there is an increasing acknowledgment of the significance of dietary factors, specifically the use of cooking oils, in contributing to health disparities [6,7]. Given that diet habits can persist for decades or even throughout life, the choice of cooking oils has become a heated topic among not only researchers but also the general public, especially considering which type of cooking oils may be more beneficial for long-term health [8].

Cooking oils, primarily used for daily food preparation, contain varying proportions of individual fatty acids along with substantial amounts of minerals and vitamins [9,10]. Cooking oils are typically derived from the extraction of plant seeds and animal fats, and the primary distinction between them lies in their fatty acid composition, with plant-based oils being rich in unsaturated fatty acids (UFAs) [9] and animal fats containing a higher proportion of saturated fatty acids (SFAs) [11]. Numerous studies have suggested that compared with animal oil, vegetable oil (including corn oil, olive oil, safflower oil, sesame oil, soybean oil, peanut oil and canola oil) may confer health benefits, such as improving insulin sensitivity, inhibiting inflammation and oxidative stress, oxidation resistance and preventing cancer cell growing [12–14]. In contrast, animal oil has been thought to be harmful to peoples’ health for years, like high consumption of SFA from animal fats is associated with a higher risk of CVD [15]. In consequence, now the dietary guidelines suggest to reduce the intake of SFAs which mainly exists in animal fats, and to increase consumption of UFAs from vegetable oil [16]. However, evidence on this topic is not entirely consistent. Ramsden found that to replace animal fats with safflower oil and safflower oil polyunsaturated margarine could increase the rate of death from cardiovascular disease (hazard ratio [HR]: 1.70,95% CI: 1.03–2.80) [17]. In another study, he found that to replace saturated fat in the diet with linoleic acid could decline serum cholesterol about 13.8% from baseline [18]. Therefore, it remains controversies that which kind of cooking oil is more beneficial to people’s health, and further research is required.

In contrast to Western countries, lard is the most prevalent animal cooking oil in China and generally regarded as a kind of unhealthy oils due to a high proportion of SFAs. In Some animal experiments, high fats lard diet was thought to induce hepatic glucose intolerance, increases prostate cancer development and progression [19,20]. Nevertheless, some studies have promoted the contrary opinion that the negative effect of lard is not that absolutely [21]. Recently, a cross-sectional study revealed that the use of lard or other animal fat oils in cooking might have greater cardiovascular health benefits among older Chinese individuals, suggesting that the dietary guidelines should carefully consider the impact of substituting vegetable or gingili oils for lard or other animal fat oils among different populations [22]. However, prospective studies on this issue are scare. In the present study, we aimed to further investigate the associations of cooking with vegetable oils and lard with survival in a nationwide, community-based, prospective cohort study of older people in China.

## 2. Methods

### 2.1. Study participants

**Fig 1:**
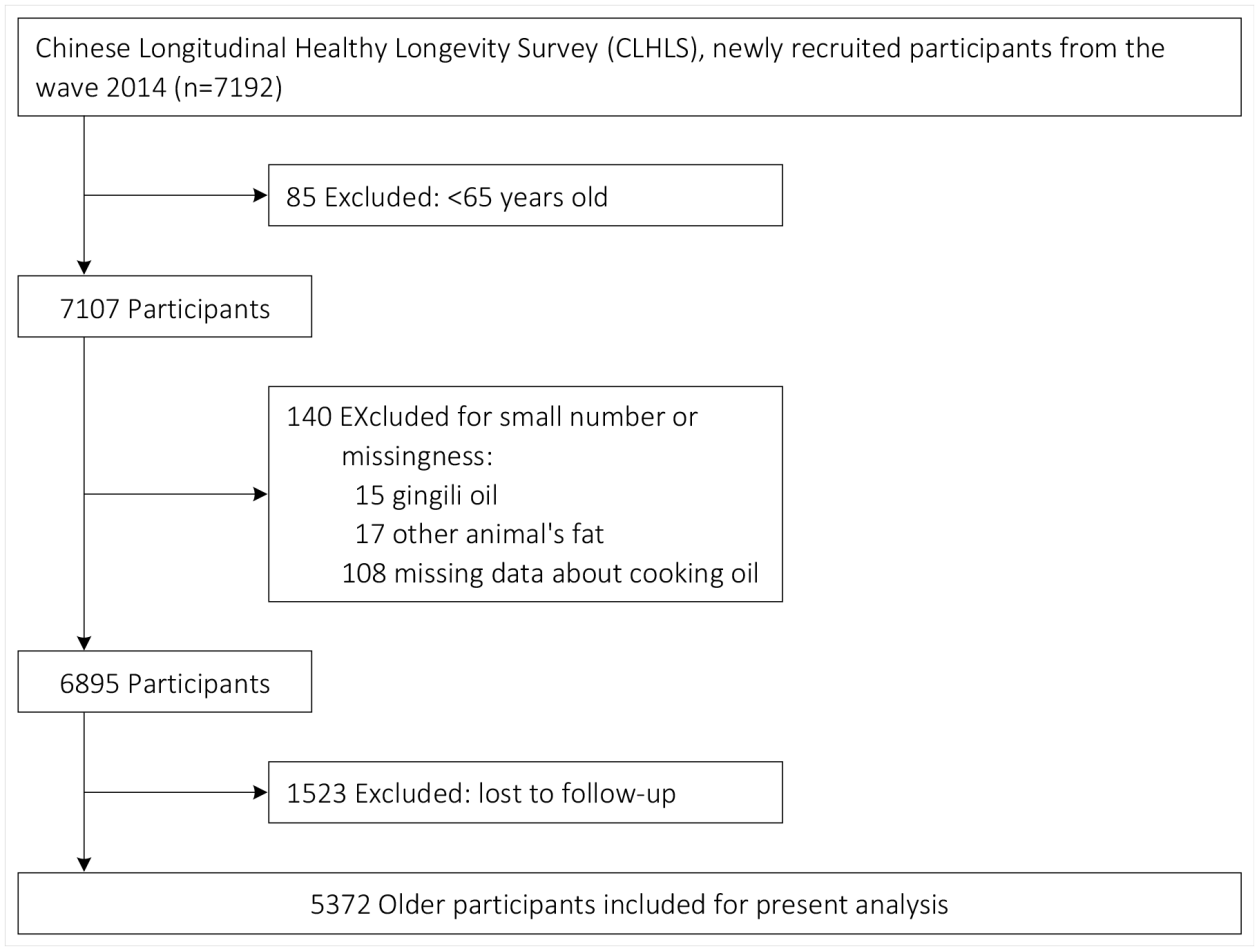
Flow chart.

The CLHLS is a nationwide, ongoing, prospective cohort study of community-dwelling older people in China, and its aim is to gain a better understanding of the determinants of healthy longevity among the elderly population. The CLHLS commenced in 1998, with subsequent follow-up conducted in 2000, 2002, 2005, 2008, 2011, 2014, and most recently in 2018. In order to mitigate attrition resulting from mortality and loss to follow-up, new participants have been enrolled during the subsequent waves since the year 2000.The CLHLS is conducted in half of the counties and cities across 23 of the 31 provinces, which are selected randomly. Approximately 85.0% of the Chinese population was covered by this survey, which was administered in participants’ homes by trained interviewers. More details about CLHLS have been reported elsewhere, with data quality generally reported as good [23–26]. The CLHLS has made significant contributions to academic research on aging, offering valuable insights that enhance long-term health and well-being in old age [27–30]. The CLHLS was conducted in accordance with the principles of the Declaration of Helsinki, and was approved by the research ethics committee of Peking University (IRB00001052–13074). Prior to participation, survey respondents provided their informed consent. Although information on cooking oils was available since the wave 2008, cause-specific mortality data were only accessible in the follow-up wave 2018 and not in the follow-up waves 2011 and 2014. Therefore, we utilized the data from the wave 2014 as the baseline, with follow-up until 2018. Fig 1 illustrates the recruitment process, and the final sample comprised 5372 older participants (aged ≥65 years).

### 2.2. Assessment of cooking oils

Types of cooking oils were evaluated through the question “What kind of grease do you mainly use for cooking?”, and the response options included vegetable oils, lard, gingili oils, and other animal’s fat. However, due to the small number of participants using gingili oils (n = 15) and other animal’s fat (n = 17), these groups were excluded from the analysis. Ultimately, based on the types of cooking oils, we categorized them into two groups: vegetable oils and lard.

### 2.3. Assessment of other covariates

S1 Table shows the detailed information of other covariates, including: sex, age, education, marital status, residence, economic income, co-residence, current smoking, current drinking, current regular exercise, regular intake of foods (fruits, vegetables, meats, fishes, eggs, and beans), comorbidities (hypertension, diabetes, heart disease, cerebrovascular disease, respiratory disease, and cancer), body mass index (BMI), waist circumference, and activities of daily living (ADL) disability. For more comprehensive information, please visit the following website: https://agingcenter.duke.edu/CLHLS.

### 2.4. All-cause and cause-specific mortality

Survival status was determined during the follow-up of 2018, including whether the participant had died, survived, or was lost to follow-up. Information regarding deaths was obtained from close family members or village doctors of the participants. Survival time was calculated from baseline to the date of death or the date of 2018 interview. Participants who could not be reached were classified as lost to follow-up. The study outcomes were overall survival (OS) and disease-specific survival (i.e., cardiovascular disease [CVD]-specific survival and non-CVD-specific survival). The International Classification of Diseases (ICD, 10th revision) was utilized to evaluate the underlying cause of death for participants. Details of the cause of death were successfully obtained for 1662 (80.5%) participants, including 433 CVD deaths (codes I00-I99) and 1229 non-CVD deaths, while 402 decedents had an unknown cause of death. Consistent with the previous literature [31], when CVD-specific survival was taken as an outcome, surviving participants were censored at the wave 2018, and the participants who died from a non-CVD cause or had an unknown cause of death were censored at their date of death. A similar methodology was employed to assess the associations between cooking oils and non-CVD-specific survival.

### 2.5. Statistical analysis

Overall, we conducted the analyses with the following steps: (1) inspections of missing values; (2) comparisons of baseline characteristics; (3) evaluating adjusted risks of cooking oils for survival; (4) performing stratified and sensitivity analyses to examine the robustness of main findings; (5) additionally exploring the associations between cooking oils and CVDs on the baseline data.

Prior to conducting data analysis, we thoroughly examined the baseline variables for any missing values. The proportion of missing data ranged from 0 to 9.07% among the variables (S2 Table). In order to enhance statistical power and minimize potential bias resulting from excluding participants with missing data from the analyses, we employed multiple imputation with chained equations to impute missing values, assuming that the data were missing at random [32]. We utilized the R mice package for imputing missing data, employing predictive mean matching, logistic regression, and polytomous regression as appropriate. Imputations were based on all baseline characteristics, along with the outcome and follow-up time. Ultimately, we generated 5 imputed datasets (5 iterations), and found no statistical differences in covariates between the complete data and the imputed data (S3 Table). All analyses except for descriptive statistics were conducted on the imputed datasets, and estimates (95% confidence interval [CI]) were combined using Rubin’s rules.

Baseline characteristics were compared between the two types of cooking oils (vegetable oils vs. lard). Continuous variables were described as median (interquartile range [IQR]), while categorical variables were presented as number (percentage). The Kruskal-Wallis test, Chi-square test, or Fisher’s exact test was employed for comparisons of baseline characteristics as appropriate. The Kaplan-Meier method was utilized to estimate the survival proportions in each group, and the log-rank test was employed for comparison. Upon utilizing the cox.zph() function in the R survival package, diagnostics revealed that cooking oils violated the proportional hazards assumption of the Cox model for OS, but did not violate the assumption for CVD-specific survival and non-CVD-specific survival. To ensure uniform presentation of results, accelerated failure time (AFT) models were subsequently used to assess the associations of cooking oils with study outcomes. AFT models estimate the time ratio (TR), which is interpreted as the expected time to events in one category relative to the reference group. Unlike interpreting proportional hazards model results where hazard ratios larger than 1 indicate higher risk, a TR greater than 1 is considered indicative of a longer time to events compared to the reference group. Based on the minimum Akaike Information Criterion among different survival distributions (e.g., weibull, lognormal, logLogistic, and gaussian), we compared the value of Akaike Information Criterion statistic of different models, and finally identified weibull distribution as most suitable for OS, lognormal distribution for CVD-specific survival and logLogistic distribution for non-CVD-specific survival, respectively (S4 Table).

Furthermore, to assess the robustness of the main findings, we performed a series of stratified and sensitivity analyses, which included: (1) conducting stratified analyses based on specific parameters. Interactions were examined by likelihood ratio testing. (2) excluding deaths that occurred within the first year or the first two years of follow-up to reduce potential reverse causation; (3) performing sensitivity analyses for participants lost to follow-up by censoring them at two time points: median and end of follow-up in order to clarify their role in the associations. (4) given that this was an observational study, it was necessary to achieve comparability of groups (vegetable oils vs. lard) with regard to potential confounding variables, and this was accomplished using propensity score matching (PSM). (5) conducting sensitivity analyses as alternatives to multiple imputation for handling missing data, such as restricting to participants with complete data or coding missing data for each variable using a missing data indicator. (6) additionally, sensitivity analyses were performed in a group excluding participants with diabetes, given its importance as a risk factor for CVD-specific survival. (7) To account for the competing risk between CVD-specific survival and non-CVD-specific survival and unknown cause of death, the Fine-Gray model was fitted to assess the associations between cooking oils and CVD-specific survival. (8) Furthermore, among the decedents, 402 (19.5%) had an unknown cause of death. The missing data may potentially result in an underestimation of the total number of CVD-specific survival or non-CVD-specific survival, and we excluded them in another sensitivity analysis. Finally, we additionally examined the associations between cooking oils and CVDs in the baseline data, and the CVDs included three available diseases (i.e., hypertension, heart disease, and cerebrovascular disease). All analyses were performed with R version 4.1.0 including the “compareGroups”, “survival”, “tidyverse”, “rms”, “mice”, “forestplot”, “survminer”, and “stats” packages (http://www.R-project.org). All tests were two sided, and p values < 0.05 were considered statistically significant.

## 3. Results

### 3.1. Baseline characteristics

Baseline characteristics of the 5372 study participants (male: 46.1%, median age: 85.0 [IQR: 77.0–93.0]) according to types of cooking oils are shown in Table 1. Compared to participants using vegetable oils, those using lard were more likely to reside in rural areas and live alone, and had lower economic income, had a lower prevalence of ADL disability and most comorbidities, had a lower proportion of regular exercise and regular intake of certain foods (i.e., fruit, vegetables, fish, eggs, and beans), and had a higher proportion of regular intake of meat. Additionally, participants using lard were more likely to have smaller BMI and waist circumference (Table 1).

**Table 1:** Baseline Characteristics.

Variables	Number	All	Vegetable oils	Lard	p value
Sex: male	5372	2474 (46.1%)	2144 (46.2%)	330 (44.9%)	0.524
Age (years)	5372	85.0 (77.0-93.0)	85.0 (77.0-93.0)	85.0 (78.0-93.0)	0.183
Education	5323				0.466
No school		3059 (57.5%)	2634 (57.3%)	425 (58.8%)	
1 year or more		2264 (42.5%)	1966 (42.7%)	298 (41.2%)	
Marital status	5308				0.140
Not in marriage		3157 (59.5%)	2709 (59.1%)	448 (62.0%)	
In marriage		2151 (40.5%)	1877 (40.9%)	274 (38.0%)	
Residence	5372				<0.001
Urban		2282 (42.5%)	2022 (43.6%)	260 (35.4%)	
Rural		3090 (57.5%)	2615 (56.4%)	475 (64.6%)	
Economic income	5300				<0.001
High		838 (15.8%)	770 (16.8%)	68 (9.4%)	
Medium or low		4462 (84.2%)	3806 (83.2%)	656 (90.6%)	
Co-residence	5332				<0.001
With family members		4162 (78.1%)	3624 (78.7%)	538 (74.1%)	
Alone		1056 (19.8%)	874 (19.0%)	182 (25.1%)	
In a nursing home		114 (2.1%)	108 (2.3%)	6 (0.8%)	
Current smoking	5350	845 (15.8%)	730 (15.8%)	115 (15.7%)	0.990
Current drinking	5324	803 (15.1%)	676 (14.7%)	127 (17.5%)	0.055
Current regular exercise	5249	1353 (25.8%)	1229 (27.1%)	124 (17.3%)	<0.001
Regular intake of foods					
Fruit	5360	2170 (40.5%)	1914 (41.4%)	256 (34.9%)	0.001
Vegetable	5361	4717 (88.0%)	4090 (88.4%)	627 (85.4%)	0.025
Meat	5327	4262 (80.0%)	3592 (78.1%)	670 (92.2%)	<0.001
Fish	5325	2621 (49.2%)	2322 (50.5%)	299 (41.1%)	<0.001
Eggs	5327	3711 (69.7%)	3359 (73.0%)	352 (48.4%)	<0.001
Beans	5323	2812 (52.8%)	2531 (55.1%)	281 (38.7%)	<0.001
Comorbidities					
Hypertension	5049	1712 (33.9%)	1542 (35.3%)	170 (25.2%)	<0.001
Diabetes	4980	278 (5.6%)	262 (6.1%)	16 (2.4%)	<0.001
Heart disease	4998	636 (12.7%)	598 (13.8%)	38 (5.7%)	<0.001
Cerebrovascular disease	5005	439 (8.8%)	418 (9.6%)	21 (3.2%)	<0.001
Respiratory disease	5029	584 (11.6%)	520 (11.9%)	64 (9.6%)	0.101
Cancer	4885	43 (0.9%)	40 (0.9%)	3 (0.5%)	0.324
BMI (kg/m^2^)	4888	21.5 (19.1-24.0)	21.7 (19.4-24.2)	20.4 (18.5-22.7)	<0.001
Waist circumference (cm)	5058	80.0 (74.0-88.0)	81.0 (74.0-89.0)	78.0 (72.0-84.0)	<0.001
ADL disability	5175	1162 (22.5%)	1074 (23.9%)	88 (13.1%)	<0.001

Values are median (IQR) or n (%). Analyses are based on complete data for each characteristic (numbers vary by characteristic and are provided in the table).

Abbreviations: ADL = activities of daily living, BMI = body mass index, IQR = inter-quartile range.

### 3.2. Association between cooking oils and survival

**Fig 2:**
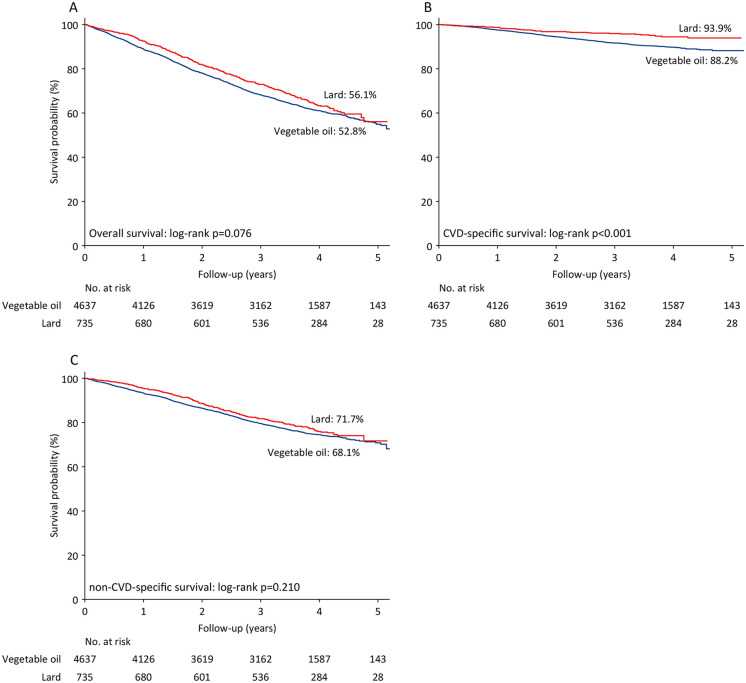
Kaplan-Meier curves for survival. Note: (A) probability of overall survival; (B) probability of CVD-specific survival; (C) probability of non-CVD-specific survival. Abbreviations: CVD = cardiovascular disease.

During a median follow-up of 3.5 years (IQR: 2.4–4.2 years), there were a total of 2064 deaths, accounting for 38.4% of the study population. Among these, there were 433 CVD deaths, 1229 non-CVD deaths, and 402 deaths with unknown causes. Kaplan-Meier analysis revealed that cooking with lard was significantly associated with a higher CVD-specific survival probability compared to cooking with vegetable oils (93.9% vs. 88.2%, log-rank p < 0.001); however, no significant differences were observed in OS and non-CVD-specific survival between the two groups (log-rank p = 0.076 and 0.210, respectively; Fig 2).

Univariate AFT models revealed a significant association between cooking with lard and longer CVD-specific survival compared to cooking with vegetable oils; however, no associations were found between cooking with lard and OS and non-CVD-specific survival. Furthermore, multivariate AFT models indicated cooking with lard was significantly associated with a 44.0% longer CVD-specific survival compared to cooking with vegetable oils (TR = 1.44, 95% CI: 1.08–1.91), and there were no associations between cooking with lard and OS and non-CVD-specific survival, as evidenced by adjusted TRs of 1.06 (95% CI: 0.95–1.18) for OS and 1.08 (95% CI: 0.93–1.26) for non-CVD-specific survival, respectively (Table 2).

**Table 2:** Associations between cooking oils and survival.

	Overall survival		CVD-specific survival		non-CVD-specific survival	
	Vegetable oil	Lard	Vegetable oil	Lard	Vegetable oil	Lard
No. of participants	4637	735	4637	735	4637	735
Deaths (n)	1796	268	399	34	1068	161
Follow-up (PYs)	14580.5	2445.7	14580.5	2445.7	14580.5	2445.7
Mortality rate (95% CI)^a^	12.3 (11.8-12.9)	11.0 (9.7-12.2)	2.7 (2.5-3.0)	1.4 (0.9-1.9)	7.3 (6.9-7.7)	6.6 (5.6-7.6)
Crude TR (95% CI), p	1.00 (ref)	1.12 (0.99-1.26), 0.069	1.00 (ref)	1.86 (1.34-2.57), < 0.001	1.00 (ref)	1.13 (0.96-1.33), 0.153
Adjusted TR (95% CI), p						
model 1^b^	1.00 (ref)	1.17 (1.05-1.31), 0.005	1.00 (ref)	1.79 (1.34-2.40), < 0.001	1.00 (ref)	1.17 (1.01-1.36), 0.042
model 2^c^	1.00 (ref)	1.19 (1.06-1.33), 0.003	1.00 (ref)	1.78 (1.32-2.39), < 0.001	1.00 (ref)	1.18 (1.01-1.38), 0.035
model 3^d^	1.00 (ref)	1.06 (0.95-1.18), 0.307	1.00 (ref)	1.44 (1.08-1.91), 0.012	1.00 (ref)	1.08 (0.93-1.26), 0.318

^a^per 100 PYs.

^b^model 1 with adjustment for sex and age.

^c^model 2 with adjustment for model 1 plus other covariates, including education, marital status, residence, economic income, co-residence, current smoking, current drinking, current regular exercise, regular intake of foods, BMI, and waist circumference.

^d^model 3 with adjustment for model 2 plus other health-related covariates, including comorbidities, and ADL disability.

Abbreviations: ADL = activities of daily living, BMI = body mass index, CI = confidence interval, CVD = cardiovascular disease, PYs = person-years, TR = time ratio.

### 3.3. Stratified and sensitivity analysis

**Fig 3:**
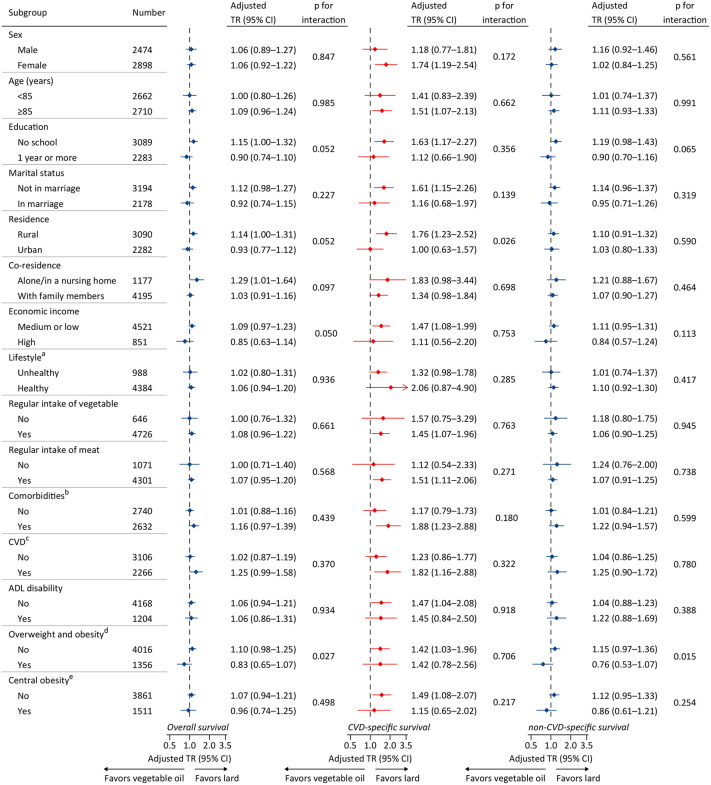
Stratified analyses by potential modifiers of the associations between cooking oil and survival. Note: ^a^ For lifestyle, a participant would be defined as “unhealthy” with meeting at least 2 of 3 following criterions, including current smoking (yes), current drinking (yes), and current regular exercise (no); otherwise, he/she was defined as “healthy”. ^b^ If a participant was with any comorbidity as shown in Table 1, he/she was defined as “yes”; otherwise, he/she was defined as “no”. ^c^ If a participant was with any comorbidity (i.e., hypertension, heart disease, and cerebrovascular disease), he/she was defined as “yes”; otherwise, he/she was defined as “no”. ^d^ Overweight and obesity was defined as a BMI ≥ 24.0 kg/m^2^ according to guidelines for a Chinese population. ^e^ Central obesity was defined as a waist circumference ≥ 90 cm in men or ≥85 cm in women. Each stratification adjusted for all factors (sex, age, education, marital status, residence, economic income, co-residence, current smoking, current drinking, current regular exercise, regular intake of foods, comorbidities, BMI, waist circumference, and ADL disability) except the stratification factor itself. The present results were obtained from imputed data set 1, and results obtained from imputations 2 to 5 were similar (data not shown). Abbreviations: ADL = activities of daily living, BMI = body mass index, CI = confidence interval, CVD = cardiovascular disease, TR = time ratio.

Stratified analyses based on participant characteristics are shown in Fig 3. The associations between cooking oils and CVD-specific survival persisted in all subgroups. Although some comparisons were not statistically significant, likely due to small sample sizes, the point estimates for longer CVD-specific survival consistently favored cooking with lard. No significant interaction was detected in all subgroups. In addition, stratified analyses generally indicated no significant associations between cooking oils and OS and non-CVD-specific survival, which is consistent with the main findings. In the sensitivity analyses, all results were similar to the main findings and the conclusions were unchanged. For example, the results remained similar after excluding deaths within the first or the first two years (S5 Table). When treating participants lost to follow-up as censored at the median or the end of follow-up, the results did not change materially (S6 Table). Similar results were also observed in the PSM sample (S7 Table, S1 Fig). Other sensitivity analyses also supported the main findings (S8 Table–S12 Tables, S2 Fig).

### 3.4. Additional analysis

We also investigated the associations between cooking oils and CVDs in the baseline data. Compared to cooking with vegetable oils, cooking with lard was significantly associated with a lower risk of CVDs (odds ratio = 0.68, 95% CI: 0.57–0.80), and cooking with lard was also found to be significantly associated with a reduced risk of the components of CVDs, including hypertension, heart disease, and cerebrovascular disease (S13 Table).

## 4. Discussion

In this study, we found that among the Chinese elderly population (aged ≥65), the use of lard as the primary cooking oil is associated with a lower risk of CVD mortality compared with vegetable oils. However, no significant association was observed for all-cause mortality or non-CVD mortality.

The primary distinction between lard and vegetable oil is in their composition, lard has a higher proportion of saturated fatty acids (SFAs) [11], while vegetable oils typically contain more unsaturated fatty acids (UFAs) [9] such as linoleic acid, oleic acid and linolenic acid. These compositional differences contribute to their different effects on human health. For recent years, dietary guidelines and public opinion have increasingly recommended to replace lard with vegetable oil, due to the evidence that some studies suggested the benefit of vegetable oil [16,33], corn oil and rapeseed oil can reduce the LDL-C (Low Density Lipoprotein Cholesterol) compared with lard [34]. However, our findings and some other studies indicate that this result is not that absolute. While some research supports the superiority of UFAs over SFAs [17,18,35], our study found that lard may have beneficial health implications. Contrarily, there exists some studies that disagree with our results. Certain studies, including a prospective analysis of 521,120 individuals aged 50–71 years from the NIH-AARP(National Institutes of Health-American Association of Retired Persons)Diet and Health Study found that intake of butter and margarine which has a high content with SFA is associated with a higher all-cause and cardiometabolic mortality, whereas oils rich in UFAs like canola and olive oil are associated with lower mortality rates [36]. The consumption of animal oils, like non-fermented milk and butter has been found to be associated with a higher overall mortality [37]. Additionally, a research involving 7888 women and 6495 men indicated that intake of SFAs and even-chain SFAs was correlated with higher total mortality in women [38]. A Systematic Review and Meta-Analysis of Prospective Cohort Studies found that there exists an inverse association between intake of linoleic acid, which is a kind of UFA, and risk of CHD, when linoleic acid replacing carbohydrates or saturated fat [39], which is also consistent with the voice that to replace saturated fat with polyunsaturated fat to prevent CHD. These studies are inconsistent with our findings that using vegetable oil as cooking oil can have a higher risk of CVD mortality.

Some studies are consistent with our findings. A review suggested that certain types of vegetable oils can inhibit vitamin K 2 -dependent processes to increase the onset of CVD [40]. A study based on The Japan Collaborative Cohort Study for Evaluation of Cancer Risk (JACC Study), which included 58,453 Japanese men and women aged 40–79 y at baseline (1988–1990) and were followed up for 14.1 y found that SFA intake and mortality from total stroke are negatively correlated [41]. Furthermore, a meta-analysis with 347,747 subjects did not find significant evidence to conclude that dietary saturated fat is associated with an increased risk of CHD or CVD [42].

Numerous studies have established that intake of SFA can increase the level of total cholesterol, and low-density lipoprotein in blood, which can increase the risk of CHD or CVD [43,44], besides, SFA trigger proinflammatory pathways and increases oxidative stress [45], which may be another cause of heart diseases. However, our findings suggest that these concerns may not fully apply to the Chinese elderly population in this cohort. Several contextual factors may be helpful to explain this discrepancy. In this study, older adults who commonly use lard as cooking oil are more likely to live in rural areas and always follow the traditional dietary patterns, which is characterized by high consumption of staple foods and vegetables, fat- soluble vitamins, cholesterol from lard may have a protective effect for older adults’ health. Besides, the proportion of different fatty acid may play a more important role in determining health outcomes than total SFAs or UFAs intake alone. However, being restricted by the data of detailed measurements of dietary nutrient intake, these mechanistic interpretations remain speculative. Further research are still needed.

There exist some limitations need to be acknowledged of this study. Firstly, the study exclusively focuses on the geriatric population in China, which limits its applicability to younger individuals. Secondly, this study groups all vegetable oils together, lack specificity regarding the types of vegetable oils used, even so, the results may still provide some insights. Thirdly, participants were classified only by their preferred type of cooking oil, but the specific amount and frequency of different types of cooking oils that participants consumed was not clearly documented. Fourthly, residual or unmeasured confounding (such as detailed dietary patterns or total nutrition intake) was still possible despite full adjustment for a wide variety of covariates related to mortality. Fifthly, the present study used information from the baseline survey and did not account for changes in cooking oils over the follow-up period. Sixthly, the data of some covariates including “current smoking”, “current drinking”, regular exercise” were all based on self-reported binary indicators, and did not have a quantitative information. Finally, our findings may have limited universality to populations from heterogeneous ethnic groups due to disparate cooking methods and prevalent types of cooking oils.

## 5. Conclusion

Overall, our findings indicate that using lard as cooking oil can be a protective factor to CVD mortality compared with vegetable oil in a majority of Chinese elder people aged 65 or above. But no significant difference for all-cause and non-CVD mortality.

## Supporting information

S1 Fig Propensity score distributional overlap and ASD. Note: In the imputed data set 1, the two groups (i.e., vegetable oil vs. lard) were matched by PSM, which was performed using the nearest neighbor matching algorithm, with a fixed caliper of 0.1 (1:2 matching, without replacement). Predicting “lard “ was modeled by multivariable logistic regression analysis, and C-index was 0.764. Figures A and B present the distributions of propensity score between the two groups in the crude and PSM samples. Area under the curve represents the probability of those propensity scores, and greater overlap of the curves indicates a lesser risk of confounding. Figure C shows ASD between the two groups in the crude and PSM samples. ASD creates a uniform scaling by which imbalance in variables may be assessed; the dashed line indicates greater than 0.100 imbalance between the variable’s values, which is a commonly used metric of significant imbalance. Results obtained from imputations 2–5 were similar (data not shown). Abbreviations: ADL = activities of daily living, ASD = Absolute standardized mean differences, BMI = body mass index, PSM = propensity score matching.
(PDF)

S2 Fig Cumulative incidence of CVD mortality (solid line) based on cooking oils and adjusted for competing risk of non-CVD mortality and unknown cause of death. (PDF)

S1 Table Definitions of baseline variables in the present study. Note: More detailed information about these covariates can be found on: https://agingcenter.duke.edu/CLHLS. Abbreviations: ADL = activities of daily living, BMI = body mass index, CLHLS = Chinese Longitudinal Healthy Longevity Surveys.
(PDF)

S2 Table Distributions of baseline variables with missing data. Note: Abbreviations: ADL = activities of daily living, BMI = body mass index.
(PDF)

S3 Table Baseline characteristics in complete data and imputed data. Note: Values are median (IQR) or n (%). ^a^Numbers vary by characteristics and are provided in the table. Abbreviations: ADL = activities of daily living, BMI = body mass index, IQR = inter-quartile range.
(PDF)

S4 Table Values of Akaike Information Criterion statistic of different distributions for survival time. Note: ^a^ Mean valus calculated from the 5 impute data sets. Abbreviations: CVD = cardiovascular disease.
(PDF)

S5 Table Association between cooking oils and mortality after excluding deaths within the first year or the first two years. Note: ^a^ With adjustment for sex, age, education, marital status, residence, economic income, co-residence, current smoking, current drinking, current regular exercise, regular intake of foods, comorbidities, BMI, waist circumference, and ADL disability. Abbreviations: ADL = activities of daily living, BMI = body mass index, CI = confidence interval, CVD = cardiovascular disease, TR = time ratio.
(PDF)

S6 Table Association between cooking oils and mortality in considering the losses censored at varying time of follow-up. Note: ^a^ With adjustment for sex, age, education, marital status, residence, economic income, co-residence, current smoking, current drinking, current regular exercise, regular intake of foods, comorbidities, BMI, waist circumference, and ADL disability. Abbreviations: ADL = activities of daily living, BMI = body mass index, CI = confidence interval, CVD = cardiovascular disease, TR = time ratio.
(PDF)

S7 Table Association between cooking oils and mortality in the PSM sample^a^. Note:^a^ Propensity score distributional overlap and ASD are shown in S1 Fig. ^b^ A separate PSM-AFT model was fitted to each of the 5 imputed data sets. To eliminate the risk of insufficient covariate balance, we further adjusted for baseline covariates in each model, including sex, age, education, marital status, residence, economic income, co residence, current smoking, current drinking, current regular exercise, regular intake of foods, comorbidities, BMI, waist circumference, and ADL disability. Finally, estimated TRs (95% CI) were combined using Rubin`s rules. Abbreviations: ADL = activities of daily living, AFT = accelerated failure model, ASD = Absolute standardized mean differences, BMI = body mass index, CI = confidence interval, CVD = cardiovascular disease, PSM = propensity score matching, TR = time ratio.
(PDF)

S8 Table Association between cooking oils and mortality among participants with complete data. Note: ^a^ With adjustment for sex, age, education, marital status, residence, economic income, co-residence, current smoking, current drinking, current regular exercise, regular intake of foods, comorbidities, BMI, waist circumference, and ADL disability. Abbreviations: ADL = activities of daily living, BMI = body mass index, CI = confidence interval, CVD = cardiovascular disease, TR = time ratio.
(PDF)

S9 Table Association between cooking oils and mortality when coding missing data using a missing data indicator^a^. Note: ^a^ For the missingness of continuous variables, BMI was categorized into overweight/obesity (yes or no) and missingness, and waist circumference was categorized into central obesity (yes or no) and missingness. ^b^ With adjustment for sex, age, education, marital status, residence, economic income, co-residence, current smoking, current drinking, current regular exercise, regular intake of foods, comorbidities, BMI, waist circumference, and ADL disability. Abbreviations: ADL = activities of daily living, BMI = body mass index, CI = confidence interval, CVD = cardiovascular disease, TR = time ratio.
(PDF)

S10 Table Association between cooking oils and mortality after excluding participants with diabetes. Note: ^a^ Values are mean (range) of the 5 imputed data sets. ^b^ With adjustment for sex, age, education, marital status, residence, economic income, co-residence, current smoking, current drinking, current regular exercise, regular intake of foods, comorbidities, BMI, waist circumference, and ADL disability. Abbreviations: ADL = activities of daily living, BMI = body mass index, CI = confidence interval, CVD = cardiovascular disease, TR = time ratio.
(PDF)

S11 Table Association between cooking oils and CVD mortality, accounting for competing risk by non-CVD mortality and unknown cause of death^a^. Note: ^a^ Given the absence of a viable method for assessing the competing risk in an accelerated failure time model, the Fine-Gray model was used here. ^b^ Non-CVD mortality and unknown cause of death were coded as event of competing risk. ^c^ With adjustment for sex, age, education, marital status, residence, economic income, co-residence, current smoking, current drinking, current regular exercise, regular intake of foods, comorbidities, BMI, waist circumference, and ADL disability. Abbreviations: ADL = activities of daily living, BMI = body mass index, CI = confidence interval, CVD = cardiovascular disease, HR = hazard ratio.
(PDF)

S12 Table Association between cooking oils and mortality after excluding participants without a documented cause of death. Note: ^a^ With adjustment for sex, age, education, marital status, residence, economic income, co-residence, current smoking, current drinking, current regular exercise, regular intake of foods, comorbidities, BMI, waist circumference, and ADL disability. Abbreviations: ADL = activities of daily living, BMI = body mass index, CI = confidence interval, CVD = cardiovascular disease, TR = time ratio.
(PDF)

S13 Table Association of cooking oils with CVD and its components (cross-sectional analysis using data from wave 2014). Note: ^a^ With adjustment for sex, age, education, marital status, residence, economic income, co-residence, current smoking, current drinking, current regular exercise, regular intake of foods, comorbidities, BMI, waist circumference, and ADL disability. ^b^ If a participant was diagnosed with any of the following diseases, including hypertension, heart disease, and cerebrovascular disease, he/she would be defined as the presence of CVD; otherwise, he/she would be defined as the absence of CVD. Abbreviations: ADL = activities of daily living, BMI = body mass index, CI = confidence interval, CVD = cardiovascular disease, OR = odds ratio.
(PDF)
