# Supplementary material for: Revisiting the associations between cooking oils and survival among older people in China: A nationwide, community-based, prospective cohort study
Source: PLoS One. 2026 Mar 5;21(3):e0344282. doi: 10.1371/journal.pone.0344282 (PMC12962501; doi:10.1371/journal.pone.0344282)
Supplement: S1 Fig — Note: In the imputed data set 1, the two groups (i.e., vegetable oil vs. lard) were matched by PSM, which was performed using the nearest neighbor matching algorithm, with a fixed caliper of 0.1 (1:2 matching, without replacement). Predicting “lard “ was modeled by multivariable logistic regression analysis, and C-index was 0.764. Figures A and B present the distributions of propensity score between the two groups in the crude and PSM samples. Area under the curve represents the probability of those propensity scores, and greater overlap of the curves indicates a lesser risk of confounding. Figure C shows ASD between the two groups in the crude and PSM samples. ASD creates a uniform scaling by which imbalance in variables may be assessed; the dashed line indicates greater than 0.100 imbalance between the variable’s values, which is a commonly used metric of significant imbalance. Results obtained from imputations 2–5 were similar (data not shown). Abbreviations: ADL = activities of daily living, ASD = Absolute standardized mean differences, BMI = body mass index, PSM = propensity score matching. (PDF) [file pone.0344282.s001.pdf]

eFigure 1. Propensity score distributional overlap and ASD

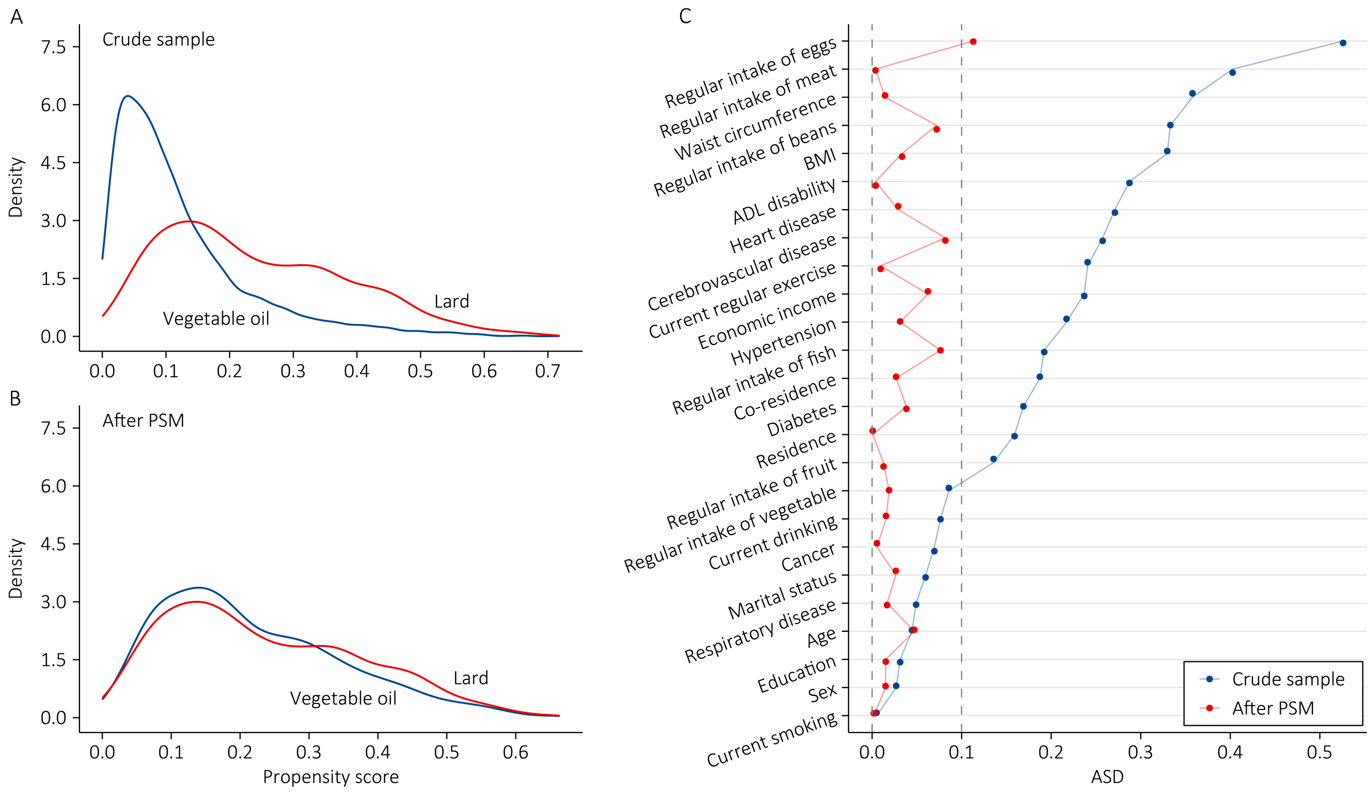

Note:

In the imputed data set 1, the two groups (i.e., vegetable oil vs. lard) were matched by PSM, which was performed using the nearest neighbor matching algorithm, with a fixed caliper of 0.1 (1:2 matching, without replacement). Predicting "lard " was modeled by multivariable logistic regression analysis, and C-index was 0.764. Figures A and B present the distributions of propensity score between the two groups in the crude and PSM samples. Area under the curve represents the probability of those propensity scores, and greater overlap of the curves indicates a lesser risk of confounding. Figure C shows ASD between the two groups in the crude and PSM samples. ASD creates a uniform scaling by which imbalance in variables may be assessed; the dashed line indicates greater than 0.100 imbalance between the variable's values, which is a commonly used metric of significant imbalance.

Results obtained from imputations 2 to 5 were similar (data not shown).

Abbreviations: ADL = activities of daily living, ASD = Absolute standardized mean differences, BMI = body mass index, PSM = propensity score matching.
