# Supplementary material for: Revisiting the associations between cooking oils and survival among older people in China: A nationwide, community-based, prospective cohort study
Source: PLoS One. 2026 Mar 5;21(3):e0344282. doi: 10.1371/journal.pone.0344282 (PMC12962501; doi:10.1371/journal.pone.0344282)

eFigure 2. Cumulative incidence of CVD mortality (solid line) based on cooking oils and adjusted for competing risk of non-CVD mortality and unknown cause of death

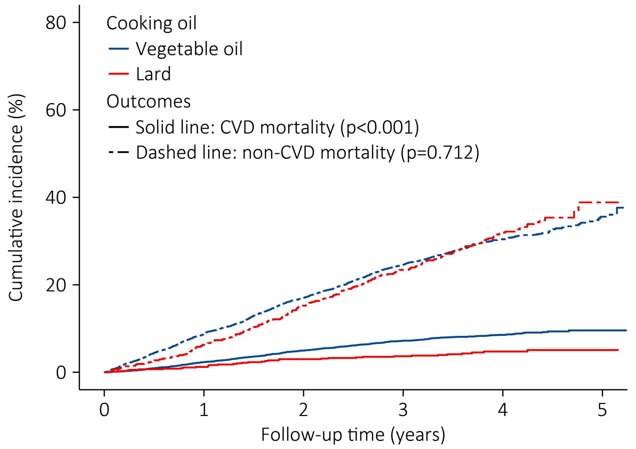

Supplement: S2 Fig — (PDF) [file pone.0344282.s002.pdf]
