# Supplementary material for: Revisiting the associations between cooking oils and survival among older people in China: A nationwide, community-based, prospective cohort study
Source: PLoS One. 2026 Mar 5;21(3):e0344282. doi: 10.1371/journal.pone.0344282 (PMC12962501; doi:10.1371/journal.pone.0344282)
Supplement: S1 Table — Note: More detailed information about these covariates can be found on: https://agingcenter.duke.edu/CLHLS. Abbreviations: ADL = activities of daily living, BMI = body mass index, CLHLS = Chinese Longitudinal Healthy Longevity Surveys. (PDF) [file pone.0344282.s003.pdf]

eTable 1. Definitions of baseline variables in the present study

Variables	Questions in the CLHLS questionnaire	Options for the questions	Reclassification in the present study
Sex		• male<br>• female	• Male<br>• Female
Age			• Continuous (years)
Education	How many years did you attend school?	• years of school<br>• don't know<br>• missing	• No school: years of school = 0<br>• 1 year or more: years of school >= 1<br>• missing: don't know, missing
Marital status	Current marital status?	• currently married and living with spouse<br>• separated<br>• divorced<br>• widowed<br>• never married<br>• don't know<br>• missing	• In marriage:<br>currently married and living with spouse, separated<br>• Not in marriage:<br>divorced, widowed, never married<br>• missing: don't know, missing
Residence	Current residence area of interviewee?	• city<br>• town<br>• rural	• Urban: city, town<br>• Rural: rural
Economic income	How do you rate your economic status compared with others in your local area?	• very rich<br>• rich<br>• so so<br>• poor<br>• very poor<br>• didn't answer<br>• missing	• High: very rich, rich<br>• Medium or low: so so, poor, very poor<br>• missing: didn`t answer, missing
Co-residence	Co-residence?	• with family member(s)<br>• alone<br>• in a nursing home<br>• missing	• With family members: with household member(s)<br>• Alone: alone<br>• In a nursing home: in a nursing home<br>• missing: missing
Current smoking	Do you smoke at present?	• yes<br>• no<br>• missing	• Current smoking: yes<br>• No smoking at present: no<br>• missing
Current drinking	Do you drink at present?	• yes<br>• no<br>• don't know<br>• missing	• Current drinking: yes<br>• No drinking at present: no<br>• missing: don't know, missing
Current regular exercise	Do you do exercises regularly at present?	• yes<br>• no<br>• don't know<br>• missing	• Current regular exercise: yes<br>• No regular exercise at present: no<br>• missing: don't know, missing

Variables	Questions in the CLHLS questionnaire	Options for the questions	Reclassification in the present study
Regular intake of fruit, and vegetable, respectively	Do you eat these foods, respectively?	• almost everyday<br>• except winter/quite often<br>• occasionally<br>• rarely or never<br>• don't know<br>• missing	• Regular intake:<br>almost everyday, except winter/quite often<br>• No regular intake:<br>occasionally, rarely or never<br>• missing: don't know, missing
Regular intake of meat, fish, eggs, and beans, respectively	Do you eat these foods, respectively?	• almost everyday<br>• not everyday, but at least once per week<br>• not every week, but at least once per month<br>• not every month, but occasionally<br>• rarely or never<br>• don't know<br>• missing	• Regular intake:<br>almost everyday; not everyday, but at least once per week<br>• No regular intake:<br>not every week, but at least once per month; not every month, but occasionally; rarely or never.<br>• missing: don't know, missing
Hypertension, diabetes, heart diseases, cerebrovascular diseases, respiratory diseases, and cancer, respectively	Are you suffering from these diseases, respectively?	• yes<br>• no<br>• don't know<br>• missing	• Yes: yes<br>• No: no<br>• missing: don't know, missing
BMI			• Continuous (kg/m^2)<br>• missing: missing; abnormal values, i.e., height < 120 cm, and weight < 25 or >= 100 kg.<br>Body weight divided by squared body height was used to compute BMI. Body weight was measured to the nearest 1 kg for participants without outer clothing, and height was measured to the nearest 1 cm for participants.
Waist circumference			• Continuous (cm)<br>• missing: missing; abnormal values, i.e., < 50 or >= 120 cm.<br>Waist circumference were measured to the nearest 1 cm using a nonelastic but flexible plastic tape. Waist circumference was measured directly between the lowest rib and iliac crest at the end of light exhalation with the participant standing with the feet touching each other.
ADL disability	Bathing:<br>without assistance?	• without assistance<br>• one part assistance<br>• more than one part assistance<br>• don't know<br>• missing	• In the CLHLS survey, six items of daily self-care ability were collected from each participant based on the Katz index: dressing, bathing, transferring, toileting, continence, and eating. Each item included three answers: complete independence, partially dependence, and complete dependence. ADL disability was defined as present if

Variables	Questions in the CLHLS questionnaire	Options for the questions	Reclassification in the present study
	Dressing: get clothes and get completely dressed without assistance?	• without assistance<br>• need assistance for trying shoes<br>• assistance in getting clothes and getting dressed<br>• missing	participants needed any assistance in performing at least one of the six self-care activities.<br>• missing: don't know, missing
	Toileting: go to the toilet, cleans self, and arranges clothes without assistance (may use object for support such as cane, walker, or wheelchair)?	• without assistance<br>• assistance in cleaning or arranging clothes<br>• don't use toilet<br>• missing	
	Transferring: get in and out of bed as well as in and out of a chair without assistance (may use object for support such as cane or walker)?	• without assistance<br>• with assistance<br>• bedridden<br>• missing	
	Continence: has complete control of urination and bowel movement without assistance?	• without assistance<br>• occasional accidents<br>• incontinent<br>• missing	
	Feeding: feed self without assistance?	• without assistance<br>• with some help<br>• need feeding<br>• missing	

More detailed information about these covariates can be found on: https://agingcenter.duke.edu/CLHLS.

Abbreviations: ADL = activities of daily living, BMI = body mass index, CLHLS = Chinese Longitudinal Healthy Longevity Surveys.
