# Supplementary material for: Revisiting the associations between cooking oils and survival among older people in China: A nationwide, community-based, prospective cohort study
Source: PLoS One. 2026 Mar 5;21(3):e0344282. doi: 10.1371/journal.pone.0344282 (PMC12962501; doi:10.1371/journal.pone.0344282)
Supplement: S2 Table — Note: Abbreviations: ADL = activities of daily living, BMI = body mass index. (PDF) [file pone.0344282.s004.pdf]

eTable 2. Distributions of baseline variables with missing data

Variables	Number of missing data	Percentage of missing data (%)
Sex	0	0.00
Age	0	0.00
Education	49	0.91
Marital status	64	1.19
Residence	0	0.00
Economic income	72	1.34
Co-residence	40	0.74
Current smoking	22	0.41
Current drinking	48	0.89
Current regular exercise	123	2.29
Regular intake of fruit	12	0.22
Regular intake of vegetable	11	0.20
Regular intake of meat	45	0.84
Regular intake of fish	47	0.87
Regular intake of eggs	45	0.84
Regular intake of beans	49	0.91
Hypertension	323	6.01
Diabetes	392	7.30
Heart diseases	374	6.96
Cerebrovascular diseases	367	6.83
Respiratory diseases	343	6.38
Cancer	487	9.07
BMI	484	9.01
Waist circumference (cm)	314	5.85
ADL disability	197	3.67

Abbreviations: ADL = activities of daily living, BMI = body mass index.
