# Supplementary material for: Revisiting the associations between cooking oils and survival among older people in China: A nationwide, community-based, prospective cohort study
Source: PLoS One. 2026 Mar 5;21(3):e0344282. doi: 10.1371/journal.pone.0344282 (PMC12962501; doi:10.1371/journal.pone.0344282)
Supplement: S3 Table — Note: Values are median (IQR) or n (%). aNumbers vary by characteristics and are provided in the table. Abbreviations: ADL = activities of daily living, BMI = body mass index, IQR = inter-quartile range. (PDF) [file pone.0344282.s005.pdf]

eTable 3. Baseline characteristics in complete data and imputed data

Variables	Complete data[a]	Imputed data sets[a]					p value
		1	2	3	4	5	
Number of participants	5372	5372	5372	5372	5372	5372	
Sex: male	2474 (46.1%)	2474 (46.1%)	2474 (46.1%)	2474 (46.1%)	2474 (46.1%)	2474 (46.1%)	1.000
Age (years)	85.0 (77.0-93.0)	85.0 (77.0-93.0)	85.0 (77.0-93.0)	85.0 (77.0-93.0)	85.0 (77.0-93.0)	85.0 (77.0-93.0)	1.000
Education							1.000
No school	3059 (57.5%)	3091 (57.5%)	3091 (57.5%)	3091 (57.5%)	3088 (57.5%)	3088 (57.5%)	
1 year or more	2264 (42.5%)	2281 (42.5%)	2281 (42.5%)	2281 (42.5%)	2284 (42.5%)	2284 (42.5%)	
Marital status							1.000
Not in marriage	3157 (59.5%)	3195 (59.5%)	3187 (59.3%)	3199 (59.5%)	3193 (59.4%)	3195 (59.5%)	
In marriage	2151 (40.5%)	2177 (40.5%)	2185 (40.7%)	2173 (40.5%)	2179 (40.6%)	2177 (40.5%)	
Residence							1.000
Urban	2282 (42.5%)	2282 (42.5%)	2282 (42.5%)	2282 (42.5%)	2282 (42.5%)	2282 (42.5%)	
Rural	3090 (57.5%)	3090 (57.5%)	3090 (57.5%)	3090 (57.5%)	3090 (57.5%)	3090 (57.5%)	
Economic income							1.000
High	838 (15.8%)	848 (15.8%)	854 (15.9%)	846 (15.7%)	848 (15.8%)	850 (15.8%)	
Medium or low	4462 (84.2%)	4524 (84.2%)	4518 (84.1%)	4526 (84.3%)	4524 (84.2%)	4522 (84.2%)	
Co-residence							1.000
With family members	4162 (78.1%)	4193 (78.1%)	4195 (78.1%)	4195 (78.1%)	4195 (78.1%)	4193 (78.1%)	
Alone	1056 (19.8%)	1064 (19.8%)	1062 (19.8%)	1062 (19.8%)	1063 (19.8%)	1065 (19.8%)	
In a nursing home	114 (2.1%)	115 (2.1%)	115 (2.1%)	115 (2.1%)	114 (2.1%)	114 (2.1%)	
Current smoking	845 (15.8%)	851 (15.8%)	849 (15.8%)	847 (15.8%)	849 (15.8%)	850 (15.8%)	1.000
Current drinking	803 (15.1%)	805 (15.0%)	809 (15.1%)	805 (15.0%)	808 (15.0%)	811 (15.1%)	1.000
Current regular exercise	1353 (25.8%)	1379 (25.7%)	1379 (25.7%)	1381 (25.7%)	1390 (25.9%)	1382 (25.7%)	1.000
Regular intake of foods							
Fruit	2170 (40.5%)	2175 (40.5%)	2176 (40.5%)	2174 (40.5%)	2178 (40.5%)	2178 (40.5%)	1.000
Vegetable	4717 (88.0%)	4727 (88.0%)	4727 (88.0%)	4727 (88.0%)	4726 (88.0%)	4727 (88.0%)	1.000
Meat	4262 (80.0%)	4293 (79.9%)	4300 (80.0%)	4301 (80.1%)	4301 (80.1%)	4296 (80.0%)	1.000
Fish	2621 (49.2%)	2644 (49.2%)	2645 (49.2%)	2646 (49.3%)	2641 (49.2%)	2642 (49.2%)	1.000
Eggs	3711 (69.7%)	3745 (69.7%)	3741 (69.6%)	3739 (69.6%)	3739 (69.6%)	3743 (69.7%)	1.000
Beans	2812 (52.8%)	2839 (52.8%)	2842 (52.9%)	2838 (52.8%)	2839 (52.8%)	2834 (52.8%)	1.000
Comorbidities							
Hypertension	1712 (33.9%)	1817 (33.8%)	1816 (33.8%)	1837 (34.2%)	1824 (34.0%)	1836 (34.2%)	0.998
Diabetes	278 (5.6%)	307 (5.7%)	318 (5.9%)	315 (5.9%)	307 (5.7%)	313 (5.8%)	0.976
Heart disease	636 (12.7%)	698 (13.0%)	703 (13.1%)	703 (13.1%)	699 (13.0%)	699 (13.0%)	0.993

Variables	Complete data[a]	Imputed data sets[a]					p value
		1	2	3	4	5	
Cerebrovascular disease	439 (8.8%)	486 (9.0%)	498 (9.3%)	490 (9.1%)	486 (9.0%)	484 (9.0%)	0.961
Respiratory disease	584 (11.6%)	638 (11.9%)	643 (12.0%)	623 (11.6%)	632 (11.8%)	631 (11.7%)	0.989
Cancer	43 (0.9%)	49 (0.9%)	48 (0.9%)	53 (1.0%)	55 (1.0%)	47 (0.9%)	0.947
BMI (kg/m^2)	21.5 (19.1-24.0)	21.4 (19.1-24.0)	21.5 (19.1-24.0)	21.5 (19.1-24.0)	21.4 (19.1-24.0)	21.5 (19.1-24.0)	0.757
Waist circumference (cm)	80.0 (74.0-88.0)	80.0 (74.0-88.0)	80.0 (74.0-88.0)	80.0 (74.0-88.0)	80.0 (74.0-88.0)	80.0 (74.0-88.0)	0.991
ADL disability	1162 (22.5%)	1205 (22.4%)	1201 (22.4%)	1208 (22.5%)	1194 (22.2%)	1204 (22.4%)	1.000

Values are median (IQR) or n (%).

[a] Numbers vary by characteristics and are provided in the table.

Abbreviations: ADL = activities of daily living, BMI = body mass index, IQR = inter-quartile range.
