# Supplementary material for: Revisiting the associations between cooking oils and survival among older people in China: A nationwide, community-based, prospective cohort study
Source: PLoS One. 2026 Mar 5;21(3):e0344282. doi: 10.1371/journal.pone.0344282 (PMC12962501; doi:10.1371/journal.pone.0344282)
Supplement: S4 Table — Note: a Mean valus calculated from the 5 impute data sets. Abbreviations: CVD = cardiovascular disease. (PDF) [file pone.0344282.s006.pdf]

eTable 4. Values of Akaike Information Criterion statistic of different distributions for survival time

Distribution	Value of Akaike Information Criterion statistic[a]
All-cause mortality	
Weibull	10935.1
Exponential	11039.6
Gaussian	11984.3
Logistic	12049.6
Lognormal	11092.4
LogLogistic	10961.3
CVD mortality	
Weibull	3625.9
Exponential	3652.7
Gaussian	3870.7
Logistic	3904.7
Lognormal	3615.9
LogLogistic	3619.6
non-CVD mortality	
Weibull	7877.8
Exponential	7934.9
Gaussian	8515.9
Logistic	8575.3
Lognormal	7944.1
LogLogistic	7874.4

[a] Mean valus calculated from the 5 impute data sets.
Abbreviations: CVD = cardiovascular disease
