# Supplementary material for: Revisiting the associations between cooking oils and survival among older people in China: A nationwide, community-based, prospective cohort study
Source: PLoS One. 2026 Mar 5;21(3):e0344282. doi: 10.1371/journal.pone.0344282 (PMC12962501; doi:10.1371/journal.pone.0344282)
Supplement: S5 Table — Note: a With adjustment for sex, age, education, marital status, residence, economic income, co-residence, current smoking, current drinking, current regular exercise, regular intake of foods, comorbidities, BMI, waist circumference, and ADL disability. Abbreviations: ADL = activities of daily living, BMI = body mass index, CI = confidence interval, CVD = cardiovascular disease, TR = time ratio. (PDF) [file pone.0344282.s007.pdf]

eTable 5. Association between cooking oils and mortality after excluding deaths within the first year or the first two years

	Excluding deaths within the first year		Excluding deaths within the first two years	
	No. of participants	Adjusted TR (95% CI)[a], p	No. of participants	Adjusted TR (95% CI)[a], p
All-cause mortality				
Vegetable oil	4806	1.00 (ref)	4220	1.00 (ref)
Lard		1.01 (0.93-1.08), 0.879		1.01 (0.95-1.07), 0.748
CVD mortality				
Vegetable oil	5256	1.00 (ref)	5120	1.00 (ref)
Lard		1.25 (1.02-1.52), 0.029		1.29 (1.09-1.53), 0.004
non-CVD mortality				
Vegetable oil	5040	1.00 (ref)	4696	1.00 (ref)
Lard		1.02 (0.93-1.13), 0.660		1.01 (0.94-1.10), 0.709

[a] With adjustment for sex, age, education, marital status, residence, economic income, co-residence, current smoking, current drinking, current regular exercise, regular intake of foods, comorbidities, BMI, waist circumference, and ADL disability.

Abbreviations: ADL = activities of daily living, BMI = body mass index, CI = confidence interval, CVD = cardiovascular disease, TR = time ratio.
