# Supplementary material for: Revisiting the associations between cooking oils and survival among older people in China: A nationwide, community-based, prospective cohort study
Source: PLoS One. 2026 Mar 5;21(3):e0344282. doi: 10.1371/journal.pone.0344282 (PMC12962501; doi:10.1371/journal.pone.0344282)
Supplement: S6 Table — Note: a With adjustment for sex, age, education, marital status, residence, economic income, co-residence, current smoking, current drinking, current regular exercise, regular intake of foods, comorbidities, BMI, waist circumference, and ADL disability. Abbreviations: ADL = activities of daily living, BMI = body mass index, CI = confidence interval, CVD = cardiovascular disease, TR = time ratio. (PDF) [file pone.0344282.s008.pdf]

eTable 6. Association between cooking oils and mortality in considering the losses censored at varying time of follow-up

	Considering the losses censored at the median of follow-up (3.49 years)		Considering the losses censored at the end of follow-up (5.24 years)	
	No. of participants	Adjusted TR (95% CI)[a], p	No. of participants	Adjusted TR (95% CI)[a], p
All-cause mortality				
Vegetable oil	6895	1.00 (ref)	6895	1.00 (ref)
Lard		0.99 (0.89-1.11), 0.907		0.96 (0.84-1.09), 0.549
CVD mortality				
Vegetable oil	6895	1.00 (ref)	6895	1.00 (ref)
Lard		1.39 (1.03-1.88), 0.032		1.43 (1.02-2.02), 0.037
non-CVD mortality				
Vegetable oil	6895	1.00 (ref)	6895	1.00 (ref)
Lard		1.02 (0.87-1.20), 0.764		1.01 (0.84-1.21), 0.906

[a] With adjustment for sex, age, education, marital status, residence, economic income, co-residence, current smoking, current drinking, current regular exercise, regular intake of foods, comorbidities, BMI, waist circumference, and ADL disability.

Abbreviations: ADL = activities of daily living, BMI = body mass index, CI = confidence interval, CVD = cardiovascular disease, TR = time ratio.
