# Supplementary material for: Revisiting the associations between cooking oils and survival among older people in China: A nationwide, community-based, prospective cohort study
Source: PLoS One. 2026 Mar 5;21(3):e0344282. doi: 10.1371/journal.pone.0344282 (PMC12962501; doi:10.1371/journal.pone.0344282)
Supplement: S7 Table — Note:a Propensity score distributional overlap and ASD are shown in S1 Fig. b A separate PSM-AFT model was fitted to each of the 5 imputed data sets. To eliminate the risk of insufficient covariate balance, we further adjusted for baseline covariates in each model, including sex, age, education, marital status, residence, economic income, co residence, current smoking, current drinking, current regular exercise, regular intake of foods, comorbidities, BMI, waist circumference, and ADL disability. Finally, estimated TRs (95% CI) were combined using Rubin`s rules. Abbreviations: ADL = activities of daily living, AFT = accelerated failure model, ASD = Absolute standardized mean differences, BMI = body mass index, CI = confidence interval, CVD = cardiovascular disease, PSM = propensity score matching, TR = time ratio. (PDF) [file pone.0344282.s009.pdf]

eTable 7. Association between cooking oils and mortality in the PSM sample[a]

	No. of participants	Adjusted TR (95% CI)[b], p
All-cause mortality		
Vegetable oil	2059	1.00 (ref)
Lard		1.06 (0.93-1.21), 0.348
CVD mortality		
Vegetable oil	2059	1.00 (ref)
Lard		1.46 (1.01-2.12), 0.047
non-CVD mortality		
Vegetable oil	2059	1.00 (ref)
Lard		1.08 (0.91-1.27), 0.373

[a] Propensity score distributional overlap and ASD are shown in eFigure 1.

[b] A separate PSM-AFT model was fitted to each of the 5 imputed data sets. To eliminate the risk of insufficient covariate balance, we further adjusted for baseline covariates in each model, including sex, age, education, marital status, residence, economic income, co-residence, current smoking, current drinking, current regular exercise, regular intake of foods, comorbidities, BMI, waist circumference, and ADL disability. Finally, estimated TRs (95% CI) were combined using Rubin`s rules.

Abbreviations: ADL = activities of daily living, AFT = accelerated failure model, ASD = Absolute standardized mean differences, BMI = body mass index, CI = confidence interval, CVD = cardiovascular disease, PSM = propensity score matching, TR = time ratio.
