# Supplementary material for: Revisiting the associations between cooking oils and survival among older people in China: A nationwide, community-based, prospective cohort study
Source: PLoS One. 2026 Mar 5;21(3):e0344282. doi: 10.1371/journal.pone.0344282 (PMC12962501; doi:10.1371/journal.pone.0344282)
Supplement: S9 Table — Note: a For the missingness of continuous variables, BMI was categorized into overweight/obesity (yes or no) and missingness, and waist circumference was categorized into central obesity (yes or no) and missingness. b With adjustment for sex, age, education, marital status, residence, economic income, co-residence, current smoking, current drinking, current regular exercise, regular intake of foods, comorbidities, BMI, waist circumference, and ADL disability. Abbreviations: ADL = activities of daily living, BMI = body mass index, CI = confidence interval, CVD = cardiovascular disease, TR = time ratio. (PDF) [file pone.0344282.s011.pdf]

eTable 9. Association between cooking oils and mortality when coding missing data using a missing data indicator[a]

	No. of participants	Adjusted TR (95% CI)[b], p
All-cause mortality		
Vegetable oil	5372	1.00 (ref)
Lard		1.04 (0.93-1.16), 0.530
CVD mortality		
Vegetable oil	5372	1.00 (ref)
Lard		1.39 (1.05-1.83), 0.021
non-CVD mortality		
Vegetable oil	5372	1.00 (ref)
Lard		1.06 (0.91-1.23), 0.477

[a] For the missingness of continuous variables, BMI was categorized into overweight/obesity (yes or no) and missingness, and waist circumference was categorized into central obesity (yes or no) and missingness.

[b] With adjustment for sex, age, education, marital status, residence, economic income, co-residence, current smoking, current drinking, current regular exercise, regular intake of foods, comorbidities, BMI, waist circumference, and ADL disability.

Abbreviations: ADL = activities of daily living, BMI = body mass index, CI = confidence interval, CVD = cardiovascular disease, TR = time ratio.
