# Supplementary material for: Revisiting the associations between cooking oils and survival among older people in China: A nationwide, community-based, prospective cohort study
Source: PLoS One. 2026 Mar 5;21(3):e0344282. doi: 10.1371/journal.pone.0344282 (PMC12962501; doi:10.1371/journal.pone.0344282)
Supplement: S10 Table — Note: a Values are mean (range) of the 5 imputed data sets. b With adjustment for sex, age, education, marital status, residence, economic income, co-residence, current smoking, current drinking, current regular exercise, regular intake of foods, comorbidities, BMI, waist circumference, and ADL disability. Abbreviations: ADL = activities of daily living, BMI = body mass index, CI = confidence interval, CVD = cardiovascular disease, TR = time ratio. (PDF) [file pone.0344282.s012.pdf]

eTable 10. Association between cooking oils and mortality after excluding participants with diabetes

	No. of participants[a]	Adjusted TR (95% CI)[b], p
All-cause mortality		
Vegetable oil	5060 (5044-5070)	1.00 (ref)
Lard		1.04 (0.92-1.16), 0.536
CVD mortality		
Vegetable oil	5060 (5044-5070)	1.00 (ref)
Lard		1.41 (1.04-1.91), 0.026
non-CVD mortality		
Vegetable oil	5060 (5044-5070)	1.00 (ref)
Lard		1.06 (0.90-1.23), 0.494

[a] Values are mean (range) of the 5 imputed data sets.

[b] With adjustment for sex, age, education, marital status, residence, economic income, co-residence, current smoking, current drinking, current regular exercise, regular intake of foods, comorbidities, BMI, waist circumference, and ADL disability.

Abbreviations: ADL = activities of daily living, BMI = body mass index, CI = confidence interval, CVD = cardiovascular disease, TR = time ratio.
