# Supplementary material for: Revisiting the associations between cooking oils and survival among older people in China: A nationwide, community-based, prospective cohort study
Source: PLoS One. 2026 Mar 5;21(3):e0344282. doi: 10.1371/journal.pone.0344282 (PMC12962501; doi:10.1371/journal.pone.0344282)
Supplement: S11 Table — Note: a Given the absence of a viable method for assessing the competing risk in an accelerated failure time model, the Fine-Gray model was used here. b Non-CVD mortality and unknown cause of death were coded as event of competing risk. c With adjustment for sex, age, education, marital status, residence, economic income, co-residence, current smoking, current drinking, current regular exercise, regular intake of foods, comorbidities, BMI, waist circumference, and ADL disability. Abbreviations: ADL = activities of daily living, BMI = body mass index, CI = confidence interval, CVD = cardiovascular disease, HR = hazard ratio. (PDF) [file pone.0344282.s013.pdf]

eTable 11. Association between cooking oils and CVD mortality, accounting for competing risk by non-CVD mortality and unknown cause of death[a]

| | Vegetable oil | Lard |
|---|---|---|
| No. of participants | 4637 | 735 |
| CVD mortality (n) | 399 | 34 |
| non-CVD mortality and unknown cause of death (n)[b] | 1397 | 234 |
| Adjusted HR (95% CI)[c], p | 1.00 (ref) | 0.62 (0.42-0.90), 0.012 |

[a] Given the absence of a viable method for assessing the competing risk in an accelerated failure time model, the Fine-Gray model was used here.

[b] Non-CVD mortality and unknown cause of death were coded as event of competing risk.

[c] With adjustment for sex, age, education, marital status, residence, economic income, co-residence, current smoking, current drinking, current regular exercise, regular intake of foods, comorbidities, BMI, waist circumference, and ADL disability.

Abbreviations: ADL = activities of daily living, BMI = body mass index, CI = confidence interval, CVD = cardiovascular disease, HR = hazard ratio.
