# Supplementary material for: Revisiting the associations between cooking oils and survival among older people in China: A nationwide, community-based, prospective cohort study
Source: PLoS One. 2026 Mar 5;21(3):e0344282. doi: 10.1371/journal.pone.0344282 (PMC12962501; doi:10.1371/journal.pone.0344282)
Supplement: S12 Table — Note: a With adjustment for sex, age, education, marital status, residence, economic income, co-residence, current smoking, current drinking, current regular exercise, regular intake of foods, comorbidities, BMI, waist circumference, and ADL disability. Abbreviations: ADL = activities of daily living, BMI = body mass index, CI = confidence interval, CVD = cardiovascular disease, TR = time ratio. (PDF) [file pone.0344282.s014.pdf]

eTable 12. Association between cooking oils and mortality after excluding participants without a documented cause of death

	No. of participants	Adjusted TR (95% CI)[a], p
All-cause mortality		
Vegetable oil	4970	1.00 (ref)
Lard		1.11 (0.97-1.26), 0.116
CVD mortality		
Vegetable oil	4970	1.00 (ref)
Lard		1.46 (1.10-1.95), 0.010
non-CVD mortality		
Vegetable oil	4970	1.00 (ref)
Lard		1.02 (0.88-1.19), 0.758

[a] With adjustment for sex, age, education, marital status, residence, economic income, co-residence, current smoking, current drinking, current regular exercise, regular intake of foods, comorbidities, BMI, waist circumference, and ADL disability.

Abbreviations: ADL = activities of daily living, BMI = body mass index, CI = confidence interval, CVD = cardiovascular disease, TR = time ratio.
