# Supplementary material for: Revisiting the associations between cooking oils and survival among older people in China: A nationwide, community-based, prospective cohort study
Source: PLoS One. 2026 Mar 5;21(3):e0344282. doi: 10.1371/journal.pone.0344282 (PMC12962501; doi:10.1371/journal.pone.0344282)
Supplement: S13 Table — Note: a With adjustment for sex, age, education, marital status, residence, economic income, co-residence, current smoking, current drinking, current regular exercise, regular intake of foods, comorbidities, BMI, waist circumference, and ADL disability. b If a participant was diagnosed with any of the following diseases, including hypertension, heart disease, and cerebrovascular disease, he/she would be defined as the presence of CVD; otherwise, he/she would be defined as the absence of CVD. Abbreviations: ADL = activities of daily living, BMI = body mass index, CI = confidence interval, CVD = cardiovascular disease, OR = odds ratio. (PDF) [file pone.0344282.s015.pdf]

eTable 13. Association of cooking oils with CVD and its components (cross-sectional analysis using data from wave 2014)

	No. of participants	Adjusted OR (95% CI)[a], p
CVD[b]		
Vegetable oil	6424	1.00 (ref)
Lard		0.68 (0.57-0.80), <0.001
Hypertension		
Vegetable oil	6438	1.00 (ref)
Lard		0.82 (0.69-0.99), 0.039
Heart disease		
Vegetable oil	6376	1.00 (ref)
Lard		0.55 (0.40-0.76), <0.001
Cerebrovascular disease		
Vegetable oil	6388	1.00 (ref)
Lard		0.50 (0.33-0.76), 0.001

[a] With adjustment for sex, age, education, marital status, residence, economic income, co-residence, current smoking, current drinking, current regular exercise, regular intake of foods, comorbidities, BMI, waist circumference, and ADL disability.

[b] If a participant was diagnosed with any of the following diseases, including hypertension, heart disease, and cerebrovascular disease, he/she would be defined as the presence of CVD; otherwise, he/she would be defined as the absence of CVD.

Abbreviations: ADL = activities of daily living, BMI = body mass index, CI = confidence interval, CVD = cardiovascular disease, OR = odds ratio.
